# Clinicoetiological Profile and Outcomes of Shock in Mechanically Ventilated Children in a Pediatric Intensive Care Unit

**DOI:** 10.7759/cureus.115664

**Published:** 2026-09-02

**Authors:** Shivali Agarwal, Ritu Rakholia, Gunjan Nagarkoti

**Affiliations:** 1 Department of Pediatrics, Government Medical College Haldwani, Haldwani, IND; 2 Department of Pediatrics, Dr. Sushila Tiwari Hospital, Government Medical College Haldwani, Haldwani, IND

**Keywords:** clinicoetiological profile, inotropes, mechanically ventilated children, mortality, pediatric age-adjusted shock index, pediatric shock, picu, septic shock, sipa

## Abstract

Introduction: Shock in mechanically ventilated children is associated with severe circulatory failure, tissue hypoperfusion, multiorgan dysfunction, prolonged intensive care requirement, and high mortality. Early bedside recognition of hemodynamic instability is essential, particularly because hypotension may appear late in pediatric shock.

Objectives: To evaluate the clinicoetiological profile and outcomes of shock in mechanically ventilated children admitted to the pediatric intensive care unit (PICU), determine the common type of shock, assess morbidity and mortality, identify factors associated with poor outcome, and determine the duration of shock reversal.

Methods: This prospective observational study included 120 children aged one month to 16 years who were admitted to the PICU over an 18-month period. Children with clinical evidence of shock requiring mechanical ventilation were enrolled by consecutive sampling after parental consent. Sociodemographic data, clinical features, vital parameters, Pediatric Age-Adjusted Shock Index (SIPA), laboratory parameters, etiological diagnosis, shock type, treatment profile, and outcomes were analyzed.

Results: Among 120 children, 63 (52.5%) survived, and 57 (47.5%) expired. Infants below one year formed the largest subgroup (40, 33.3%), and males predominated (77, 64.2%). Septic shock was the most common shock type (104, 86.7%), and respiratory infection/pneumonia/acute respiratory distress syndrome was the leading etiology (48, 40.0%). Younger age, decompensated shock, shock duration >4 hours, blood pressure below the 5th centile, higher mean SIPA, altered sensorium, pallor, cyanosis, edema, thrombocytopenia, deranged coagulation, raised transaminases, hyperlactatemia, requirement of multiple inotropes, transfusion requirement, and mechanical ventilation >72 hours were significantly associated with mortality.

Conclusion: Septic shock secondary to severe respiratory infection was the predominant etiology and major contributor to mortality among mechanically ventilated pediatric shock patients. Hemodynamic instability, persistent hypoperfusion, organ dysfunction, and prolonged intensive care support were important markers of poor outcome. Early recognition using bedside hemodynamic assessment, including SIPA, protocol-based resuscitation, timely vasoactive therapy, and aggressive management of sepsis may improve survival.

## Introduction

Pediatric shock is an acute state of circulatory failure in which oxygen delivery becomes insufficient to meet tissue metabolic demand. The resulting shift from aerobic to anaerobic metabolism leads to lactic acidosis, cellular dysfunction, and progressive organ injury if timely resuscitation is not instituted. Contemporary pediatric sepsis and shock definitions emphasize tissue hypoperfusion and organ dysfunction rather than hypotension alone, because children may maintain blood pressure until late stages of circulatory failure [1].

Children differ from adults in their cardiovascular response to shock. Tachycardia, increased myocardial contractility, and peripheral vasoconstriction can temporarily preserve systemic blood pressure despite compromised perfusion. Hence, hypotension in children is usually a late and ominous sign, and reliance on blood pressure alone may delay diagnosis and escalation of therapy [1,2].

Shock in the pediatric intensive care unit (PICU) may be septic, hypovolemic, cardiogenic, obstructive, or distributive. Septic shock remains the dominant clinical subtype in many pediatric intensive care settings and is frequently precipitated by pneumonia, severe systemic infection, or central nervous system infection. The pathophysiology involves vasodilation, capillary leak, endothelial dysfunction, myocardial depression, and impaired microcirculatory oxygen utilization [2,3].

Children with shock who require invasive mechanical ventilation represent a particularly high-risk subgroup. Ventilatory support is often required due to respiratory failure, altered consciousness, severe metabolic acidosis, or refractory hemodynamic compromise. Positive pressure ventilation itself may influence venous return and cardiac output, thereby adding complexity to hemodynamic management in preload-dependent states [3].

The burden of pediatric septic shock and critical illness remains high in low- and middle-income countries. Delayed referral, advanced disease at presentation, severe infectious burden, and limited access to advanced monitoring contribute to higher mortality compared with well-resourced settings [4,5]. Indian tertiary care and government hospital PICUs frequently manage critically ill children referred late from peripheral facilities, making early bedside risk stratification clinically important.

Simple hemodynamic tools, such as the Shock Index and Pediatric Age-Adjusted Shock Index (SIPA) [6], may help identify children at risk of deterioration using readily available vital parameters. SIPA score is a useful tool for rapid detection of shock, traditionally used for hypovolemic shock in trauma patients. This, however, can be used for triage and risk stratification in emergency settings as it has fewer parameters and is easy to implement. This score is particularly useful where rapid laboratory or invasive hemodynamic monitoring is not immediately available [7]. The present study was undertaken to evaluate the clinicoetiological profile and short-term outcomes of shock in mechanically ventilated children admitted to a PICU and to identify clinical, laboratory, and management-related predictors of mortality.

## Materials and methods

This was a prospective observational study conducted in the PICU of Government Medical College, Haldwani, a tertiary care teaching hospital that caters to the Kumaon areas of Uttarakhand and adjoining areas of Uttar Pradesh. The study was conducted over a period of 18 months after approval of the Institutional Ethics Committee.

Study population

The study population comprised critically ill children aged one month to 16 years with clinical evidence of shock requiring mechanical ventilation and admitted to the PICU. Consecutive sampling of eligible patients whose parents/guardians gave written informed consent was undertaken. A total of 120 children with clinical evidence of shock on mechanical ventilation were evaluated. The following sample size formula was used for sample size calculation:

$N = \frac{z^2pq}{d^2}$

Where Z = 1.96, p = 8.6%, q = 91.4%, and d = 5.

$N = \frac{1.96 \times 1.96 \times 8.6 \times 91.4}{5 \times 5} = 120$

Operational definition of shock

Shock was considered when a child fulfilled at least one of the following: tachycardia in a normothermic state, blood pressure below the 5th centile, weak or absent distal pulses, core-peripheral temperature gap, or capillary refill time more than three seconds.

Shock was further classified as compensated (state in which blood pressure is maintained above the 5th centile despite reduced tissue perfusion) and decompensated (state in which hypotension is present, that is, blood pressure is less than the 5th centile and progressive tissue injury develops). Shock was classified on the basis of etiology as cardiogenic (cardiac pump failure or myocardial dysfunction), hypovolemic (decreased preload secondary to internal or external losses), obstructive (decreased cardiac output secondary to direct impediment to right or left heart outflow), distributive (abnormalities of vasomotor tone from loss of venous and arterial capacitance), and septic (decreased tissue perfusion following microbial infection).

Inclusion criteria

Children aged one month to 16 years admitted to the PICU with clinical evidence of shock on mechanical ventilation whose parents gave written informed consent.

Exclusion criteria

Children below one month and above 16 years of age. Patients with clinical evidence of shock who were not on mechanical ventilation.

Data collection

Sociodemographic details, past history, immunization status, presenting symptoms, vital parameters, systemic examination findings, laboratory investigations, arterial blood gas/lactate, etiological diagnosis, type and severity of shock, fluid bolus requirement, inotrope requirement, transfusion requirement, and mechanical ventilation duration were recorded in a structured proforma.

SIPA assessment

SIPA was calculated from heart rate and systolic blood pressure and assessed as a bedside hemodynamic marker wherever age-specific reference values were available [6].

Treatment and outcome assessment

Children were managed as per institutional PICU protocol, including establishing two wide-bore peripheral vascular access, fluid resuscitation with Ringer's lactate or normal saline with 10-20 ml/kg fluid bolus up to 60 ml/kg, vasoactive support with epinephrine and non-epinephrine, antibiotics, and ventilator support. Outcomes were recorded as survivor or non-survivor, duration of shock, shock reversal, duration of mechanical ventilation, morbidity profile, and mortality pattern.

Statistical analysis

Categorical variables were expressed as frequency and percentage, and continuous variables as mean ± standard deviation. Outcome-wise comparisons were performed using the chi-square test or Fisher's exact test for categorical variables and independent t-test for continuous variables. A p-value <0.05 was considered statistically significant.

## Results

The study included 120 mechanically ventilated children with shock, of whom 63 (52.5%) survived and 57 (47.5%) expired. The largest age group was infants below one year (40, 33.3%), followed by one to three years (26, 21.7%). Males were more common (77, 64.2%) than females (43, 35.8%). Lower middle socioeconomic status was the most common category (60, 50.0%). Fever (99, 82.5%), shortness of breath (98, 81.7%), and altered sensorium (87, 72.5%) were the most frequent presenting symptoms. Septic shock was the predominant type of shock (104, 86.7%), and respiratory infection/pneumonia/acute respiratory distress syndrome (ARDS) was the most common etiology (48, 40.0%).

Figure 1 shows that respiratory infection/pneumonia/ARDS was the most common etiological category, accounting for 48 children (40.0%), followed by central nervous system (CNS)/neurological infection or disorder in 25 children (20.8%) and septicemia/systemic sepsis in 12 children (10.0%). The etiological distribution highlights the predominance of severe infective respiratory and neurological illnesses in this mechanically ventilated pediatric shock cohort.

**Figure 1:**
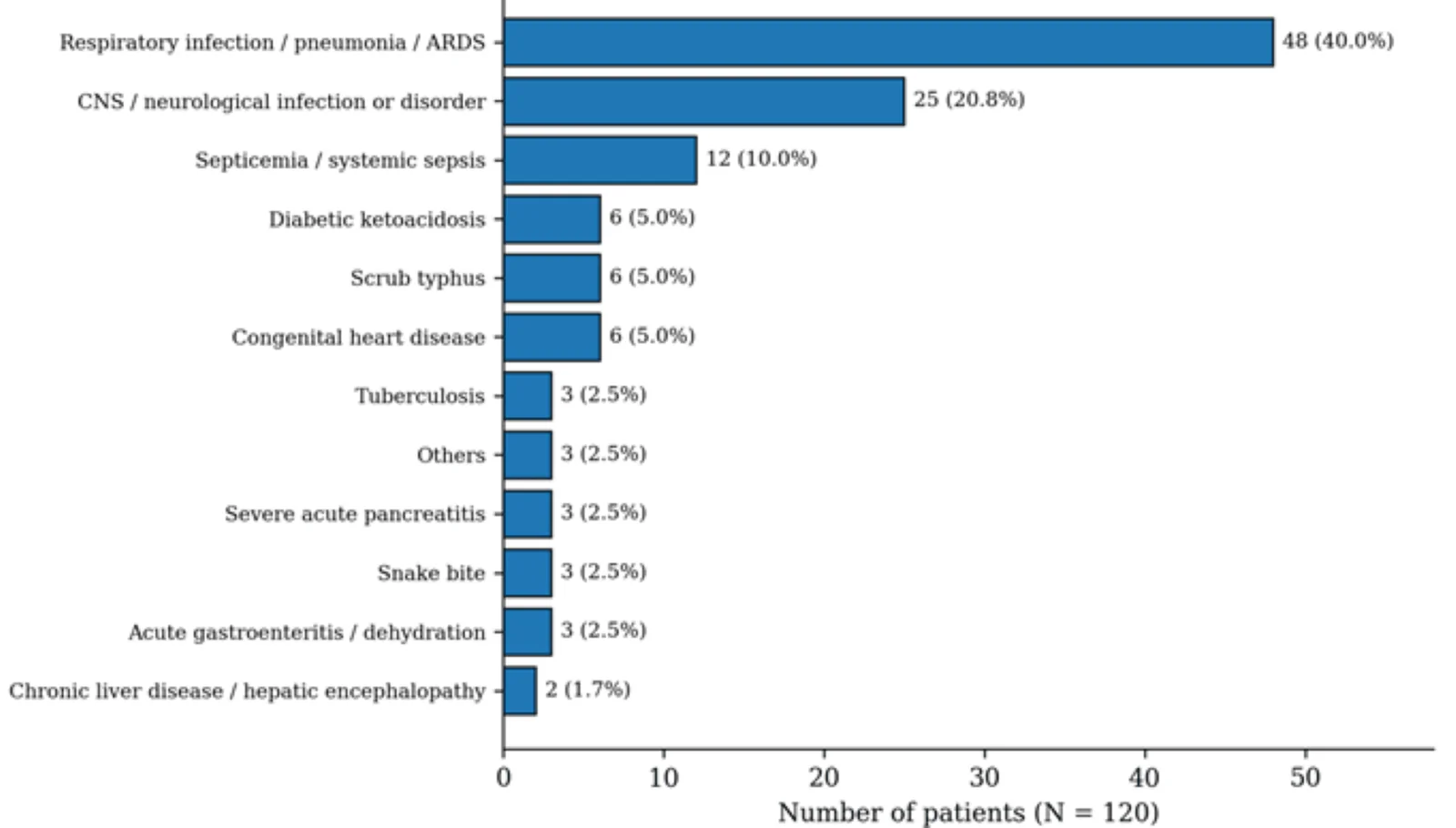
Etiological profile of shock among study participants (N = 120). ARDS: acute respiratory distress syndrome; CNS: central nervous system.

In Table 1, infants below one year formed the largest subgroup and contributed a higher proportion of deaths (25, 43.9%) than survivors (15, 23.8%), with a significant association between age group and outcome (p = 0.001). Gender and socioeconomic status were not significantly associated with outcome. Past-history distribution showed a significant outcome-wise association (p = 0.045), and incomplete immunization was more frequent among non-survivors (16, 28.1%) than survivors (6, 9.5%) (p = 0.017).

**Table 1:** Sociodemographic and background profile according to outcome status. Statistical test: Chi-square test. CVS: cardiovascular system; CNS: central nervous system.

Variable	Category	Survivors (n = 63)	Non-survivors (n = 57)	Total (N = 120)	p-value
Age group	<1 year	15 (23.8%)	25 (43.9%)	40 (33.3%)	0.001
	1-3 years	12 (19.0%)	14 (24.6%)	26 (21.7%)	
	4-6 years	15 (23.8%)	0 (0.0%)	15 (12.5%)	
	7-10 years	3 (4.8%)	5 (8.8%)	8 (6.7%)	
	11-13 years	9 (14.3%)	9 (15.8%)	18 (15.0%)	
	14-16 years	9 (14.3%)	4 (7.0%)	13 (10.8%)	
Gender	Male	45 (71.4%)	32 (56.1%)	77 (64.2%)	0.120
	Female	18 (28.6%)	25 (43.9%)	43 (35.8%)	
Socioeconomic status	Upper middle	24 (38.1%)	21 (36.8%)	45 (37.5%)	0.441
	Lower middle	29 (46.0%)	31 (54.4%)	60 (50.0%)	
	Lower	10 (15.9%)	5 (8.8%)	15 (12.5%)	
Past history	Insignificant	27 (42.9%)	28 (49.1%)	55 (45.8%)	0.045
	CVS: heart disease	6 (9.5%)	0 (0.0%)	6 (5.0%)	
	CNS: hydrocephalus	3 (4.8%)	6 (10.5%)	9 (7.5%)	
	Respiratory: bronchial asthma	3 (4.8%)	0 (0.0%)	3 (2.5%)	
	Other significant past history	24 (38.1%)	23 (40.4%)	47 (39.2%)	
Immunization history	Complete	57 (90.5%)	41 (71.9%)	98 (81.7%)	0.017
	Incomplete	6 (9.5%)	16 (28.1%)	22 (18.3%)	

Figure 2 shows that septic shock was the predominant type of shock (104, 86.7%), followed by cardiogenic shock (8, 6.7%). Decompensated shock was present in 84 children (70.0%), while shock duration greater than four hours was documented in 102 children (85.0%), indicating advanced circulatory compromise at presentation in most patients. The study included critically ill, mechanically ventilated children; hence, organ system involvement was common in both groups. Multiple organ dysfunction syndrome (MODS) was present in a higher percentage of survivors (100% versus 87.7%), as some patients had a fulminant course, probably not allowing time for organ dysfunction to manifest. This should not be interpreted as evidence that organ dysfunction was protective.

**Figure 2:**
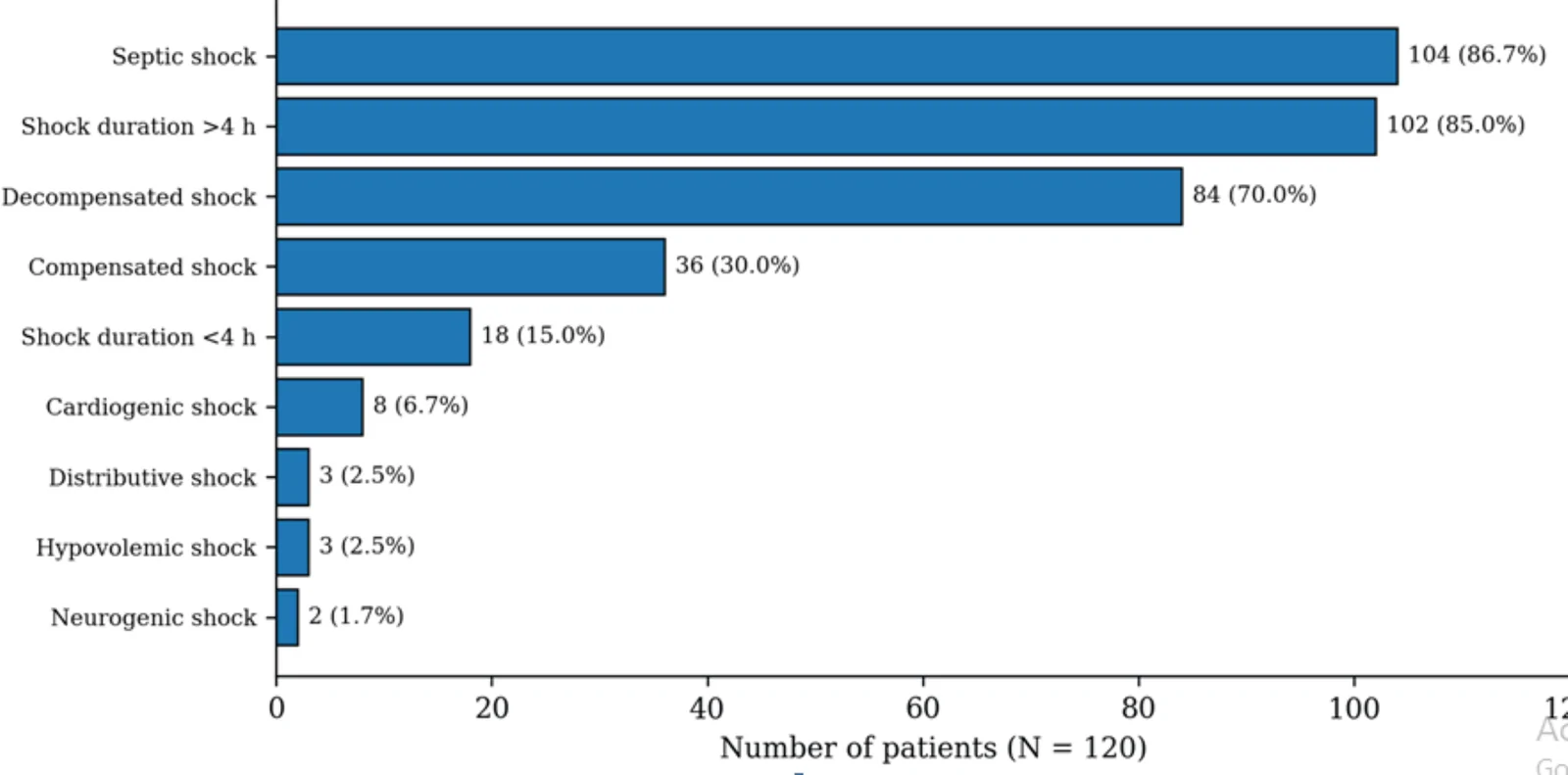
Type, severity, and duration of shock among study participants (N = 120).

In Table 2, fever and altered sensorium were significantly more frequent among non-survivors. Non-survivors had significantly higher mean heart rate and SIPA index, with significantly lower systolic and diastolic blood pressure. Blood pressure below the 5th centile was seen in 56 (98.2%) non-survivors compared with 27 (42.9%) survivors (p < 0.001). Pallor, cyanosis, edema, and organ-system involvement were also significantly associated with outcome.

**Table 2:** Clinical, hemodynamic, and SIPA profile according to outcome status. Statistical tests: Chi-square test/Fisher's exact test for categorical variables and independent t-test for continuous variables. * Fisher's exact test was used where cell counts were small. BP: blood pressure; SIPA: Pediatric Age-Adjusted Shock Index; MODS: multiple organ dysfunction syndrome.

Parameter	Category/value	Survivors (n = 63)	Non-survivors (n = 57)	Total (N = 120)	p-value
Fever	Present	45 (71.4%)	54 (94.7%)	99 (82.5%)	0.002
Shortness of breath	Present	54 (85.7%)	44 (77.2%)	98 (81.7%)	0.333
Altered sensorium	Present	36 (57.1%)	51 (89.5%)	87 (72.5%)	<0.001
Heart rate (/min)	Mean ± SD	144.90 ± 28.76	155.61 ± 24.49	149.99 ± 27.24	0.030
Respiratory rate (/min)	Mean ± SD	50.38 ± 16.90	48.35 ± 24.70	49.42 ± 20.90	0.604
Systolic BP (mmHg)	Mean ± SD	87.24 ± 13.12	76.32 ± 10.22	82.05 ± 12.99	<0.001
Diastolic BP (mmHg)	Mean ± SD	50.98 ± 9.00	42.49 ± 9.19	46.95 ± 10.00	<0.001
SIPA index	Mean ± SD	1.75 ± 0.44	2.04 ± 0.61	1.89 ± 0.55	0.004
Blood pressure centile	BP <5th centile	27 (42.9%)	56 (98.2%)	83 (69.2%)	<0.001
	BP >=5th centile	36 (57.1%)	1 (1.8%)	37 (30.8%)	
SIPA status	Abnormal SIPA	42 (66.7%)	25 (43.9%)	67 (55.8%)	0.421
	Normal SIPA	6 (9.5%)	7 (12.3%)	13 (10.8%)	
	Reference not available	15 (23.8%)	25 (43.9%)	40 (33.3%)	-
Pallor	Present	15 (23.8%)	30 (52.6%)	45 (37.5%)	0.002
Cyanosis	Present	3 (4.8%)	16 (28.1%)	19 (15.8%)	0.001
Edema	Present	0 (0.0%)	9 (15.8%)	9 (7.5%)	0.001*
MODS/organ involvement	Present	63 (100.0%)	50 (87.7%)	113 (94.2%)	0.013

In Table 3, non-survivors had significantly lower mean hemoglobin and platelet count, with significantly higher prothrombin time, international normalized ratio (INR), serum glutamic oxaloacetic transaminase (SGOT), serum glutamic pyruvic transaminase (SGPT), and lactate levels. The higher lactate level among non-survivors indicates more severe tissue hypoperfusion. Total leukocyte count, creatinine, and C-reactive protein (CRP) did not show statistically significant outcome-wise differences.

**Table 3:** Laboratory parameters according to outcome status. Statistical test: Independent t-test. INR: international normalized ratio; SGOT: serum glutamic oxaloacetic transaminase; SGPT: serum glutamic pyruvic transaminase; CRP: C-reactive protein; mm^3^: cubic millimeter.

Laboratory parameter	Survivors (n = 63)	Non-survivors (n = 57)	Overall mean ± SD	p-value
Hemoglobin (g/dL)	10.81 ± 3.26	8.99 ± 1.78	9.95 ± 2.81	<0.001
Total leukocyte count (/mm^3^)	13,861.90 ± 6,860.86	12,385.96 ± 14,752.55	13,160.83 ± 11,291.15	0.492
Platelet count (/mm^3^)	229,976.19 ± 201,277.79	137,000.00 ± 122,773.60	185,812.50 ± 174,283.34	0.003
Prothrombin time (seconds)	16.62 ± 2.42	23.58 ± 16.31	19.93 ± 11.85	0.002
INR	1.31 ± 0.20	2.28 ± 0.94	1.78 ± 0.82	<0.001
SGOT (IU/L)	103.10 ± 122.80	231.96 ± 415.38	164.31 ± 305.33	0.028
SGPT (IU/L)	64.48 ± 76.10	170.74 ± 305.47	114.95 ± 223.09	0.013
Creatinine (mg/dL)	2.10 ± 6.97	1.49 ± 1.01	1.81 ± 5.09	0.491
Lactate (mmol/L)	2.28 ± 1.02	3.19 ± 1.94	2.71 ± 1.59	0.002
CRP (mg/L)	47.33 ± 43.15	47.31 ± 60.81	47.32 ± 52.06	0.998

In Table 4, CNS/neurological etiologies were disproportionately represented among non-survivors. Septic shock, decompensated shock, shock duration >4 hours, fluid bolus volume >40 ml/kg, requirement of more than one inotrope, prolonged inotrope support, transfusion requirement, and mechanical ventilation >72 hours were significantly associated with mortality. The present study shows that higher fluid bolus administration of more than 40 ml/kg was associated with increased mortality. This is in concurrence with the recent Surviving Sepsis Guidelines 2026, which, in response to the FEAST trial, highlight the potential risk of increased mortality with fluid bolus therapy in critically ill children in resource-limited settings. Children requiring larger volumes may have had more severe or refractory shock, and therefore higher fluid administration may partly represent a marker of illness severity rather than a direct cause of mortality. Our findings support a judicious, response-guided approach to fluid resuscitation rather than avoidance of fluid therapy altogether [8,9].

**Table 4:** Etiological, shock-related, and management predictors according to outcome status. Statistical tests: Chi-square test/Fisher's exact test as applicable. ARDS: acute respiratory distress syndrome; CNS: central nervous system.

Parameter	Category	Survivors (n = 63)	Non-survivors (n = 57)	Total (N = 120)	p-value
Etiology	Respiratory infection/pneumonia/ARDS	24 (38.1%)	24 (42.1%)	48 (40.0%)	<0.001
	CNS/neurological infection or disorder	3 (4.8%)	22 (38.6%)	25 (20.8%)	
	Septicemia/systemic sepsis	9 (14.3%)	3 (5.3%)	12 (10.0%)	
	Congenital heart disease	6 (9.5%)	0 (0.0%)	6 (5.0%)	
	Diabetic ketoacidosis	3 (4.8%)	3 (5.3%)	6 (5.0%)	
	Scrub typhus	6 (9.5%)	0 (0.0%)	6 (5.0%)	
Type of shock	Septic shock	51 (81.0%)	53 (93.0%)	104 (86.7%)	0.045
	Cardiogenic shock	6 (9.5%)	2 (3.5%)	8 (6.7%)	
	Other shock types	6 (9.5%)	2 (3.5%)	8 (6.7%)	
Severity of shock	Compensated	36 (57.1%)	0 (0.0%)	36 (30.0%)	<0.001
	Decompensated	27 (42.9%)	57 (100.0%)	84 (70.0%)	
Duration of shock	<4 hours	18 (28.6%)	0 (0.0%)	18 (15.0%)	<0.001
	>4 hours	45 (71.4%)	57 (100.0%)	102 (85.0%)	
Fluid bolus volume	<40 ml/kg	57 (90.5%)	42 (73.7%)	99 (82.5%)	0.029
	>40 ml/kg	6 (9.5%)	15 (26.3%)	21 (17.5%)	
Inotrope requirement	No inotrope	12 (19.0%)	0 (0.0%)	12 (10.0%)	<0.001
	One inotrope	36 (57.1%)	6 (10.5%)	42 (35.0%)	
	More than one inotrope	15 (23.8%)	51 (89.5%)	66 (55.0%)	
Inotrope support duration	>24 hours	39 (61.9%)	57 (100.0%)	96 (80.0%)	<0.001
Transfusion requirement	Required	27 (42.9%)	40 (70.2%)	67 (55.8%)	0.005
Mechanical ventilation duration	<72 hours	27 (42.9%)	3 (5.3%)	30 (25.0%)	<0.001
	24-72 hours	0 (0.0%)	3 (5.3%)	3 (2.5%)	
	>72 hours	36 (57.1%)	51 (89.5%)	87 (72.5%)	

## Discussion

The present prospective observational study evaluated 120 mechanically ventilated children with shock admitted to a PICU. The overall mortality was 47.5%, indicating that shock requiring mechanical ventilation represents an advanced and high-risk stage of pediatric critical illness. Infants below one year formed the largest age group and contributed the highest proportion of deaths, suggesting increased vulnerability of younger children to rapid hemodynamic deterioration and organ dysfunction.

Male predominance was observed in the study population; however, gender did not show a statistically significant association with mortality. Age group was significantly associated with outcome, with a larger proportion of non-survivors among infants. Similar vulnerability of younger children has been reported in pediatric septic shock and mechanically ventilated cohorts, where immature physiological reserve, severe infection burden, and delayed presentation may contribute to adverse outcomes [10-12].

Septic shock was the predominant type of shock, accounting for 86.7% of cases, and respiratory infection/pneumonia/ARDS was the leading etiology. Respiratory and CNS/neurological etiologies together constituted the majority of mortality. These findings are clinically relevant in Indian tertiary care PICUs, where severe pneumonia, meningoencephalitis, systemic sepsis, and delayed referral remain common drivers of critical illness. Similar patterns have been reported in resource-limited pediatric intensive care settings, where respiratory failure and sepsis are major contributors to PICU mortality [5,13].

Clinical variables strongly reflected outcome. Fever and altered sensorium were significantly more frequent among non-survivors, and altered sensorium showed a particularly strong association with mortality. Pallor, cyanosis, edema, and organ-system involvement were also more common in fatal cases, suggesting that systemic hypoperfusion, tissue hypoxia, and multiorgan dysfunction were important determinants of poor outcome. These bedside findings remain important in settings where advanced monitoring may not be immediately available.

Hemodynamic parameters showed clear outcome-wise differences. Non-survivors had significantly higher heart rate and mean SIPA index, with significantly lower systolic and diastolic blood pressure. Blood pressure below the 5th centile was present in 98.2% of non-survivors. Although categorical SIPA status was not significantly associated with outcome, mean SIPA was significantly elevated among non-survivors, supporting its value as a bedside marker of hemodynamic instability. Recent studies have similarly highlighted SIPA as a simple and practical tool for early risk stratification in pediatric sepsis and PICU patients [7,14].

Management-related variables also reflected disease severity. Children requiring fluid bolus volume >40 ml/kg, more than one inotrope, prolonged inotrope support, transfusion, and mechanical ventilation for more than 72 hours had significantly higher mortality. Prolonged mechanical ventilation and vasoactive support have been associated with increased morbidity, resource utilization, and death in pediatric intensive care cohorts [15,16]. These findings emphasize the need for early resuscitation, careful fluid strategy, timely vasoactive support, and continuous reassessment of perfusion response [10].

Laboratory abnormalities among non-survivors included anemia, thrombocytopenia, prolonged PT, raised INR, elevated transaminases, and higher lactate levels. These abnormalities indicate impaired oxygen delivery, coagulation dysfunction, hepatic involvement, tissue hypoperfusion, and multiorgan dysfunction. Hyperlactatemia was significantly higher among non-survivors, supporting the role of lactate as a marker of ongoing anaerobic metabolism and inadequate perfusion in shock states. Similar Indian pediatric sepsis studies have also identified laboratory abnormalities, multiorgan dysfunction, and requirement of supportive care as important predictors of progression and poor outcome [17].

The present study has some limitations. It was conducted at a single tertiary care center with a sample size of 120 and included only children requiring mechanical ventilation; therefore, results may not represent the full spectrum of pediatric shock. Advanced hemodynamic monitoring, Pediatric Logistic Organ Dysfunction-2 (PELOD-2) scoring, detailed microbiological correlation, and long-term neurodevelopmental outcomes were not assessed, although PELOD-2 has been reported as a useful mortality prediction tool in pediatric intensive care settings [18]. SIPA reference values were unavailable for infants below one year, limiting uniform age-wise SIPA interpretation. Due to the lack of multivariate analysis, we were unable to identify independent predictors of mortality. Despite these limitations, the study provides clinically useful evidence regarding outcome predictors in a high-risk mechanically ventilated pediatric shock population.

## Conclusions

The present study demonstrated that shock among mechanically ventilated pediatric patients is associated with substantial morbidity and mortality. Septic shock was the predominant shock type, and respiratory infection/pneumonia/ARDS was the leading etiology, followed by CNS/neurological infection or disorder and systemic sepsis. Infants and younger children formed a major high-risk subgroup. Mortality was significantly associated with younger age, decompensated shock, shock duration greater than four hours, hypotension, higher mean SIPA, altered sensorium, pallor, cyanosis, edema, multiorgan dysfunction, thrombocytopenia, deranged coagulation, elevated transaminases, hyperlactatemia, multiple inotrope requirement, prolonged inotropic support, transfusion requirement, and mechanical ventilation beyond 72 hours. Bedside clinical assessment and simple hemodynamic indices such as SIPA may support early identification of high-risk children. Early recognition of shock progression, protocol-based resuscitation, judicious fluid administration, timely vasoactive therapy, and aggressive treatment of sepsis and organ dysfunction are essential for improving survival in mechanically ventilated pediatric shock patients.

## References

[1] Schlapbach LJ, Watson RS, Sorce LR (2024). International consensus criteria for pediatric sepsis and septic shock. JAMA.

[2] Garcia PCR, Tonial CT, Piva JP (2020). Septic shock in pediatrics: the state-of-the-art. J Pediatr (Rio J).

[3] Lee EP, Wu HP, Chan OW, Lin JJ, Hsia SH (2022). Hemodynamic monitoring and management of pediatric septic shock. Biomed J.

[4] de Souza DC, Machado FR (2019). Epidemiology of pediatric septic shock. J Pediatr Intensive Care.

[5] Assies R, Snik I, Kumwenda M (2022). Etiology, pathophysiology and mortality of shock in children in low (middle) income countries: a systematic review. J Trop Pediatr.

[6] Kinjalk M, Jain N, Neogi S, Ratan SK, Panda SS, Sehgal M, Arora V (2024). Pediatric Age-adjusted Shock Index (SIPA): from injury to outcome in blunt abdominal trauma. J Indian Assoc Pediatr Surg.

[7] Tanveer A, Gowa MA, Nawaz H, Chandio B, Jamal G, Bibi N (2025). Role of shock index and pediatric age adjusted shock index as a predictor of mortality and morbidity in patients admitted at PICU. Professional Med J.

[8] Maitland K, Kiguli S, Opoka RO (2011). Mortality after fluid bolus in African children with severe infection. N Engl J Med.

[9] Weiss SL, Peters MJ, Oczkowski SJW (2026). Surviving Sepsis Campaign international guidelines for the management of sepsis and septic shock in children 2026. Pediatr Crit Care Med.

[10] Ranjit S, Natraj R (2024). Hemodynamic assessment and management of septic shock in children. J Pediatr Crit Care.

[11] Monorika PG, Biswas G, Ghosh C (2025). Risk factors associated with mortality in pediatric septic shock: a single centre experience from Eastern India. Eur J Cardiovasc Med.

[12] Gadappa SM, Behera MK (2018). Clinical profile and outcome of shock in mechanically ventilated patients in pediatric intensive care unit of tertiary care centre. Int J Res Med Sci.

[13] Bacha AJ, Gadisa DA, Gudeta MD, Beressa TB, Negera GZ (2023). Survival status and predictors of mortality among patients admitted to pediatric intensive care unit at selected tertiary care hospitals in Ethiopia: a prospective observational study. Clin Med Insights Pediatr.

[14] Rehman A, Sarwar M, Abbas S, Sultana N, Jamil A, Pervez A (2025). Pediatric age-adjusted shock index in sepsis: a predictor of mortality in the intensive care unit of a tertiary care hospital. Pak J Intens Care Med.

[15] Kawaguchi A, Fernandez A, Baudin F (2025). Prevalence, management, health-care burden, and 90-day outcomes of prolonged mechanical ventilation in the paediatric intensive care unit (LongVentKids): an international, prospective, cross-sectional cohort study. Lancet Child Adolesc Health.

[16] Zhang Z, Cai X, Ming M (2024). Incidence, outcome, and prognostic factors of prolonged mechanical ventilation among children in Chinese Mainland: a multi-center survey. Front Pediatr.

[17] Shah S, Deshmukh CT, Tullu MS (2020). The predictors of outcome and progression of pediatric sepsis and septic shock: a prospective observational study from western India. J Postgrad Med.

[18] Deshmukh T, Varma A, Damke S, Meshram R (2020). Predictive efficacy of Pediatric Logistic Organ Dysfunction-2 score in pediatric intensive care unit of rural hospital. Indian J Crit Care Med.

